# Can ELABELA be a novel target in the treatment of chronic lymphocytic leukaemia?

**DOI:** 10.1186/s12885-019-6325-6

**Published:** 2019-11-12

**Authors:** Didar Yanardag Acik, Mehmet Bankir, Filiz Alkan Baylan, Bilal Aygun

**Affiliations:** 1 Department of Internal Medicine and Haematology, Adana City Education and Research Hospital, Mithat Özsan Bulvarı Kışla Mah. 4522 Sok. No:1, 01260 Yüreğir, Adana, Turkey; 2 Department of Internal Medicine, Adana City Training and Research Hospital, Mithat Özsan Bulvarı Kışla Mah. 4522 Sok. No:1, 01260 Yüreğir, Adana, Turkey; 3 Department of Biochemistry, Kahramanmaraş Sütçü İmam University Faculty of Medicine, Mithat Özsan Bulvarı Kışla Mah. 4522 Sok. No:1, 01260 Yüreğir, Adana, Turkey; 4 Department of Internal Medicine and Haematology, Adana City Education and Research Hospital, Mithat Özsan Bulvarı Kışla Mah. 4522 Sok. No:1, 01260 Yüreğir, Adana, Turkey

**Keywords:** ELABELA, Apelinergic system, Chronic lymphocytic leukaemia, Apoptosis

## Abstract

**Background:**

It has been shown that bcl2, bcl-XL and mcl-1 protein levels are high in chronic lymphocytic leukemia cells, and resultantly, apoptosis does not occur chronic lymphocytic leukemia cells. Apelin and apela (ELABELA/ELA/Toddler) are two peptide ligands for a class A G-protein coupled receptor called apelin receptor. Studies have shown that ELA inhibits apoptosis by inhibiting apoptotic proteins and activating anti-apoptotic proteins. Proteins and genes involved in apoptosis are valuable for targeted cancer therapy. We hypothesized that serum levels may be increased in patients with chronic lymphocytic leukemia based on the antiapoptotic effect of ELA. We compared serum ELABELA levels of healthy volunteers and patients with chronic lymphocytic leukemia. We aimed to draw attention to a new molecule worthy of research in targeted cancer treatment.

**Methods:**

Forty two untreated CLL patients and 41 healthy volunteers were included in the study. Serum ELA levels were measured by using enzyme-linked immunosorbent assay kits (Dhanghai Sunred Biological Technology co. Ltd), automated ELISA reader (Thermo Scientific, FİNLAND) and computer program (Scanlt for Multiscan F.C.2.5.1) in accordance with the manufacturer’s instructions. Statistical analysis was done by Statistical Package for Social Sciences for Windows 20 (IBM SPSS Inc., Chicago, IL) ve MedCalc programs. ELA and variables related to CLL were correlated with Spearman correlation anlysis test. ROC analysis and Youden index method were used to determine a cut off point for ELA. All *p*-values were 2-sided with statistical significance at 0.05 alpha levels.

**Results:**

In our study, we found that serum ELA levels were significantly higher in patients with CLL.

**Conclusions:**

This study highlights that ELA targeting may be a potential therapeutic option for treating CLL.

## Background

Chronic lymphocytic leukaemia (CLL) is the most frequent type of leukaemia in adults worldwide [1]. It is a malignancy characterised by accumulation of small, neoplastic CD5^+^ B cells with a mature appearance in blood, bone marrow and secondary lymphoid tissues, lymphadenopathy and splenomegaly [2]. In contrast to malignant cells of other B lymphocytes, the majority of CLL cells are arrested in the G0/G1 cell transformation phase because they do not possess proliferative capacity. Therefore, CLL does not occur as a result of excessive B cell proliferation but because of defective apoptosis [3]. The mechanism of apoptosis is complex and involves two separate regulatory pathways: the intrinsic and extrinsic pathways. The intrinsic pathway is regulated by the bcl-2 family. Bcl-2 itself is an anti-apoptotic protein and is part of a complex including MCL-1, BCL-XL, BCL-W and BFL-1, all of which support cell survival. The bcl-2 family members, including BAX and BAK, which are homo-oligomerized when activated and regulate outer mitochondrial membrane permeability, cause irreversible caspase activation and subsequently apoptotic cell death [4].

Studies have suggested that bcl 2, bcl-XL and mcl-1 protein levels are high in CLL cells, and therefore, apoptosis does not occur in CLL cells [3, 4].

The clinical course of CLL considerably varies. This variability has been linked to mutations in *TP53*. *TP53* is the most important predictor of response to therapy and prognosis. It has been found that *TP53* undergoes mutation in half of all human cancer cases and that loss of regulatory function of *TP53* leads to oncogenesis. Loss of function of *TP53* is considered to be an important event in tumour formation and is also associated with chemotherapy resistance and poor prognosis in many cancers [5].

Therefore, mechanisms that activate or inhibit TP53 have been the focus of research in targeted cancer therapy.

Apelin and ELA are two peptide ligands for a class A G-protein coupled receptor called apelin receptor (AR/APJ/APLNR). These ligands function by binding to this receptor; this is known as the apelinergic system (Apelin/APJ system). The binding of both endogenous peptides to AR results in similar physiological effects [6]. It is well known that the Apelin/APJ system can regulate apoptosis in various cell types and subsequently mediate the formation and development of related diseases. Recent evidence suggests that the Apelin/APJ system affects apoptosis in various diseases through different signalling pathways. Pre-treatment of cardiomyocytes with apelin-13 effectively inhibits apoptosis caused by glucose withdrawal and can significantly increase Akt and mTOR phosphorylation by upregulating Bcl-2 and downregulating Bax and cleaved caspase-3 expression. The Apelin/APJ system also upregulates the expression of Bcl-2 and downregulates the expression of Bax protein [7–11].

ELA (also known as Ende, Elabela and Toddler) was first identified in a gene expression panel for new mouse endoderm-specific genes and is evolutionally conserved among vertebrates. In zebrafish, loss of ELA disrupts mesendodermal cell movement during gastrulation, resulting in defects in endoderm differentiation and heart development and in posterior malformations. ELA acts as an endogenous ligand for APLNR, its G-protein-linked receptor, and ELA and APLNR have been shown to direct angioblast migration to control the vascular pattern in zebrafish embryos [12–14]. ELA is highly expressed in human blastocysts prior to implantation and contributes to the pluripotency of human embryonic stem cells (hESCs) via an alternative receptor [15].

The non-coding region of ELA has been shown to play a role in the regulation of apoptosis induced by p53-mediated DNA damage in mouse embryonic stem cells. ELA downregulates the interaction between heterogeneous nuclear ribonucleoprotein L (hnRNPL) and p53 [16].

In this study, we aimed to investigate the relationship between ELA and CLL because the apelinergic system blocks the caspase system that induces apoptosis and validate the anti-apoptotoic effects of ELA that have been demonstrated in previous studies.

## Methods

This prospective study was approved by the ethics committee, and 42 patients diagnosed between 2012 and 2019 at Adana Numune Training and Research Hospital and followed up without treatment and 41 healthy controls were evaluated. Written informed consent was obtained from patients and healthy volunteers. The diagnosis of CLL was made according to iw CLL [17] and the patients were staged according to Rai staging system [18]. The data included gender; age; white blood cell count (WBC); lymphocyte count; hemoglobin (Hb) level; platelet count; presence of Del13q14, p53. Blood samples were drawn from the subjects and centrifuged at 4000 rpm for 10 min and stored at − 80 ^0^ C as serum until use. Serum ELA levels were measured by by using enzyme-linked immunosorbent assay (ELISA) kits (Dhanghai Sunred Biological Technology co. Ltd), automated ELISA reader (Thermo Scientific, FİNLAND) and computer program (Scanlt for Multiscan F.C.2.5.1) in accordance with the manufacturer’s instructions. Sensitivity was 0,118 ng/ml and assay rangewas 0,15 ng/ml – 40 ng/ml. Intra-Assay %CV was <%10 and inter- assay %CV was <%12’dir. The results were expressed as ng/ml.

### Statistical analysis

Statistical analysis was made by Statistical Package for Social Sciences (SPSS) for Windows 20 (IBM SPSS Inc., Chicago, IL) ve MedCalc programs. The normality of the data was evaluated by Kolmogorov-Smirnov test. Data were described as numbers and percentage or median and range or mean ± standart deviation, when appropriate. T test (for normally distrubeted data) and Mann Whitney U test for continuous values to campare the numeric values between the patient and control groups. x^2^ Fisher’s exact test was used for evaluating categorical values. ELA and variables related to CLL were correlated with Spearman correlation anlysis test. ROC analysis and Youden index method were used to determine a cut off point for ELA. All *p*-values were 2-sided with statistical significance at 0.05 alpha levels.

## Results

The study population comprised 83 subjects: 41 in the control group and 42 with CLL. There was no significant difference between the CLL and control group in mean age (63.9 ± 9.8 vs. 61.7 ± 10.2, *P* = 0.332). The ratio of male patients was higher in the CLL group than in the control group (66.7% vs. 39%, *P* = 0.016) (Table 1).

**Table 1:** Demographic and laboratory findings in the control and CLL groups

Variables	Entire population *N* = 83	CLL group *N* = 42	Control group *N* = 41	p
Age (years)	62.8 ± 10.0	63.9 ± 9.8	61.7 ± 10.2	0.326
Gender				
Female	39 (47.0)	14 (33.3)	25 (61.0)	0.016*
Male	44 (53.0)	28 (66.7)	16 (39.0)	
Haemoglobin (g/dL)	12.6 ± 2.1	12.5 ± 2.2	12.7 ± 2.0	0.707
WBCs (×10^3^/μL)	13.7 (2.6–131)	27.1 (5.8–131)	7.7 (2.6–16.3)	< 0.001*
Neutrophils (×10^3^/μL)	4.7 (0.6–11.7)	5.5 (0.6–11.5)	4.6 (1.9–11.7)	0.078
Lymphocytes (×10^3^/μL)	5 (0.4–123)	21.3 (5–123)	2.1 (0.4–4.1)	< 0.001*
Platelets (×10^3^/μL)	225 (24–630)	200 (24–463)	253 (49–630)	0.008*
ELABELA (ng/ml)	4.6 (0.1–19.7)	6.7 (0.6–19.7)	2 (0.1–8.6)	< 0.001*

Numerical variables are presented as mean ± standard deviation or median (min-max) according to normality distribution. Categorical variables are presented as number (%)

* *P* < 0.05 indicates statistical significance

Abbreviations: *CLL* Chronic lymphocytic leukaemia, *WBC* White blood cell

**Fig. 1:**
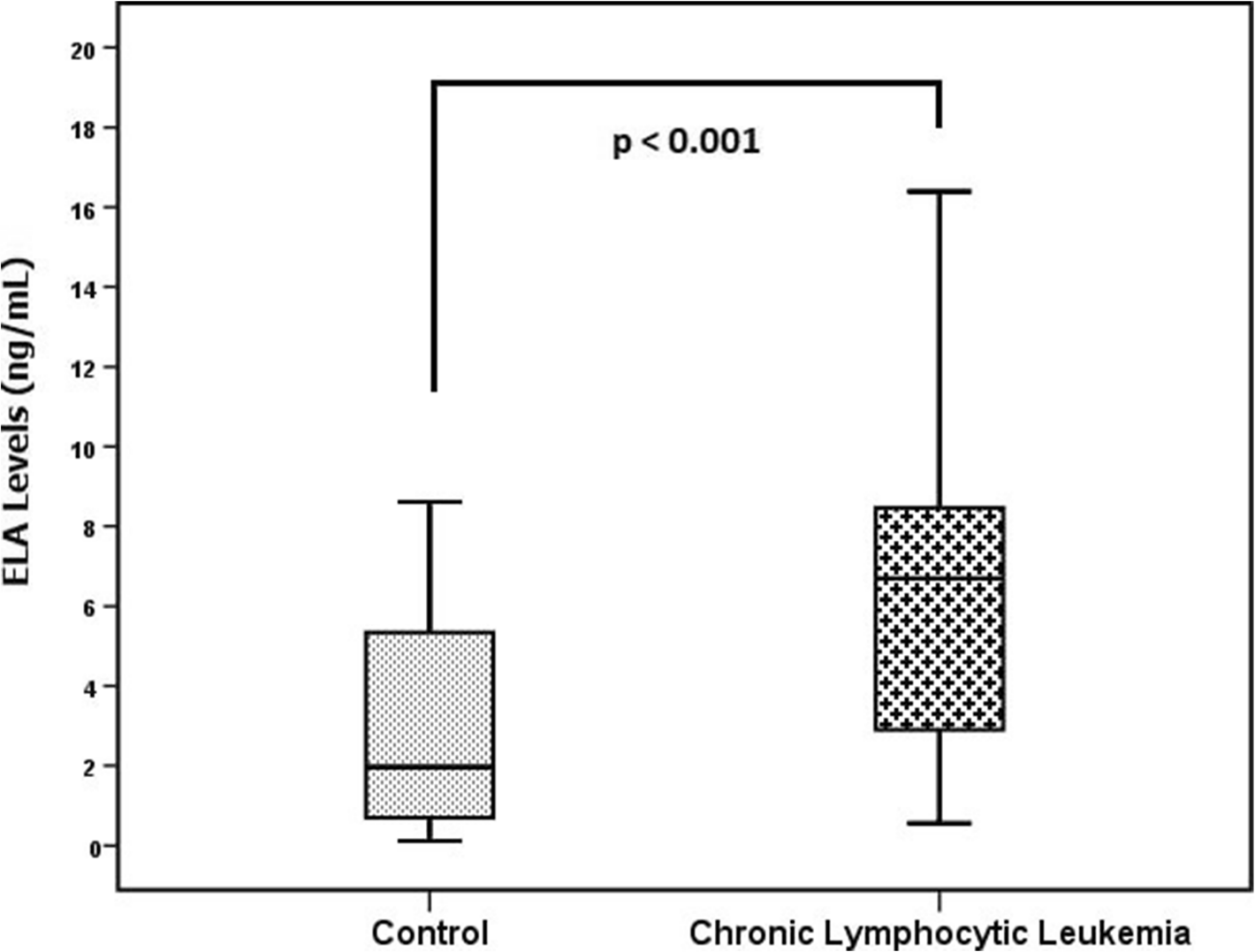
Mean ELA levels in the control and CLL groups

There was no significant difference between the CLL and control group in mean haemoglobin levels (12.5 ± 2.2 g/dL vs. 12.7 ± 2 g/dL, *P* = 0.707) and median neutrophil levels (5.5 × 10^3^/μL vs. 4.6 × 10^3^/μL, *P* = 0.078). However, median white blood cell (WBC) count (27.1 × 10^3^ cells/μL vs. 7.7 × 10^3^ cells/μL, *P* < 0.001), median lymphocyte count (21.3 × 10^3^ cells/μL vs. 2.1 × 10^3^ cells/μL, P < 0.001) and median ELA levels (6.7 ng/ml vs. 2 ng/ml, P < 0.001) were found to be higher in the CLL group than in the control group (Fig. 1), whereas the median platelet level was lower in the CLL group than in the control group (200 × 10^3^/μL vs. 253 × 10^3^/μL, *P* = 0.008) (Table 1).

In the control and CLL groups, the ELA level did not exhibit a significant correlation with gender and age (Table 2).

**Table 2:** ELABELA levels according to demographic findings in patients with CLL

Group	Variables	ELABELA (ng/ml)	p
CLL	Gender		
	Female	8.6 (0.6–19.7)	0.062
	Male	6.3 (0.6–11)	
	Age	r = 0.056	0.728
Control	Gender		
	Female	3.5 (0.2–8.6)	0.234
	Male	1.3 (0.1–7.0)	
	Age	r = −0.003	0.985

ELABELA levels is presented as median (min-max)

r = Spearman’s correlation coefficient

Abbreviations: *CLL* Chronic lymphocytic leukaemia

The disease duration was 2–84 months in the CLL group, and the median disease duration was 24 months. Further, 23.8% of the patients (*n* = 10) had stage 2, 14.3% (*n* = 6) had stage 3 and 7.1% (*n* = 3) had stage 4 disease. The direct coombs (DC) test was performed in all patients with CLL, and 11.9% (*n* = 5) were positive. The p53 test was performed in 21 patients, and 28.6% (n = 6) were positive. The del13q test was performed in 14 patients, and 64.3% (*n* = 9) were positive.

Among patients with CLL, ELA levels did not significantly differ according to the disease stage and between patients with positive and negative DC test results, patients with positive and negative p53 test results and between patients with positive and negative delq13 test results (Table 3).

**Table 3:** ELABELA levels according to disease stage and tests in patients with CLL

Variables	CLL	ELABELA (ng/ml)	p
STAGE	N = 42		
0	7 (16.7)	7.6 (6.2–19.6)	0.361
1	16 (38.1)	6.3 (0.6–19.7)	
2	10 (23.8)	6.3 (0.6–9.7)	
3	6 (14.3)	6.4 (0.7–15.8)	
4	3 (7.1)	4.8 (1.1–7.3)	
DC	N = 42		
Negative	34 (81.0)	7 (0.6–19.7)	0.134
Positive	5 (11.9)	2.9 (0.7–6.9)	
Weak positive	3 (7.1)	3 (1.1–19.6)	
p53	*N* = 21		
Negative	15 (71.4)	7.6 (0.7–19.7)	0.080
Positive	6 (28.6)	3.4 (0.6–8.8)	
DEL13q	*N* = 14		
Negative	5 (35.7)	6.2 (0.7–9.7)	0.898
Positive	9 (64.3)	6.6 (0.6–16.4)	

ELABELA level is presented as median (min-max). Categorical variables are presented as number (%)

Abbreviations: *CLL* Chronic lymphocytic leukaemia

**Fig. 2:**
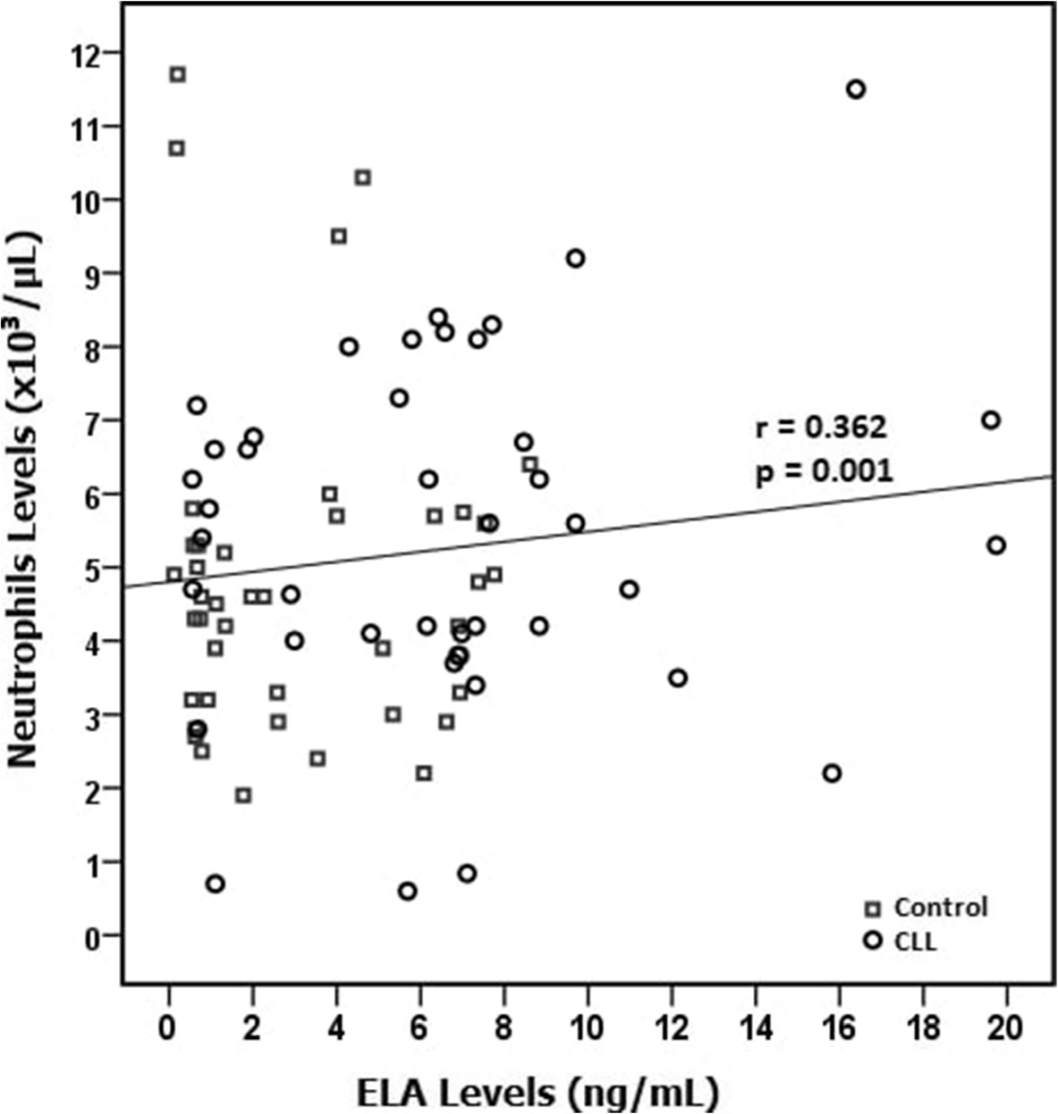
Relationship between ELA levels and neutrophil levels

There was a positive correlation between ELA levels and WBC count (r = 0.357, *P* = 0.001) and lymphocyte count (r = 0.362, P = 0.001) in the study population (Fig. 2). No correlation was found between ELAlevels and other laboratory findings. In patients with CLL, there was no significant relationship between ELA levels and disease duration, stage and laboratory findings (Table 4).

**Table 4:** Clinical findings related to ELABELA levels in patients with CLL

Variables	Study population		CLL	
	ELABELA		ELABELA	
	r	p	r	p
Disease duration	–	–	−0.153	0.332
Stage	–	–	−0.246	0.116
Haemoglobin	0.050	0.655	0.087	0.582
WBC	0.357	0.001*	0.087	0.585
Neutrophil	0.091	0.411	0.051	0.748
Lymphocyte	0.362	0.001*	0.068	0.667
Platelet	−0.202	0.067	−0.041	0.797

r = Spearman’s correlation coefficient

Abbreviations: *WBC* White blood cell

In the multivariate logistic regression model, gender, WBC count, platelet levels and ELA levels were found to be associated with CLL. Furthermore, WBC count and ELA levels were identified as independent risk factors for predicting CLL (WBC: OR = 1.58, *P* < 0.001; ELA: OR = 1.38, P < 0.001) (Table 5).

**Table 5:** Risk factors for CLL

Variables	Univariate			Multivariate		
	OR	95% CI	p	OR	95% CI	p
Gender						
Female	ref					
Male	3.13	1.27–7.67	0.013*			
WBC	1.61	1.29–2.00	< 0.001*	1.58	1.26–2.01	< 0.001*
Neutrophil	1.13	0.93–1.37	0.229			
Platelet	0.98	0.97–0.98	0.026*			
ELABELA	1.31	1.13–1.52	< 0.001*	1.38	1.17–1.63	< 0.001*
				Nagelkerke R^2^ = 0.849; p < 0.001*		

Abbreviations: *OR* Odds ratio, *CI* Confidence intervals

* *P* < 0.05 indicates statistical significance

**Fig. 3:**
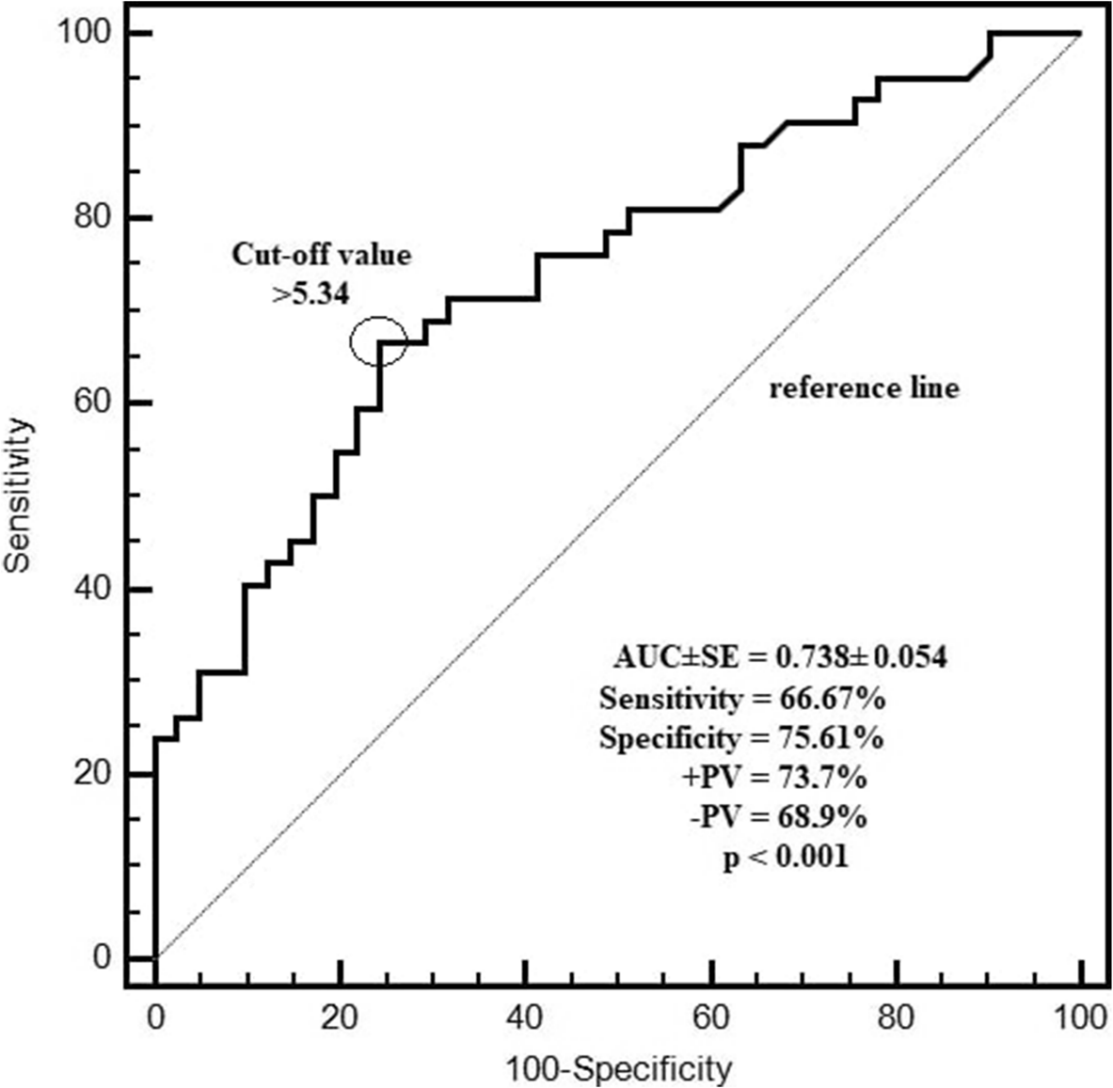
Evaluation of the diagnostic performance of ELA level in predicting CLL

The cut-off value for WBC in predicting CLL was found to be > 13.9, with 92.9% sensitivity and 97.6% specificity (+PV: 97.5%, −PV: 93%, AUC ± SE = 0.965 ± 0.023, P < 0.001). The cut-off value for ELA level in predicting CLL was found to be > 5.34, with 66.7% sensitivity and 75.6% specificity (+PV: 73.7%, −PV: 68.9%, AUC ± SE = 0.738 ± 0.054, P < 0.001) (Fig. 3).

In patients with CLL, the ratio of DC-negative patients was found to be higher in patients with an ELA level > 5.34 (ng/ml) compared with those with an ELA level of ≤5.34 (92.9% vs. 57.1%, *P* = 0.011). In patients with CLL, there was no significant relationship between patients with an ELA level > 5.34 (ng/ml) and patients with an ELA level of ≤5.34 (ng/ml) in demographic parameters and other clinical findings (Table 6).

**Table 6:** Demographic characteristics and clinical findings according to the cut-off ELABELA levels in predicting CLL

Variables	ELABELA (ng/ml)		p
	≤5.34 N = 14	> 5.34 *N* = 28	
Age (years)	63.7 ± 8.3	63.9 ± 10.6	0.948
Gender			
Female	4 (28.6)	10 (35.7)	0.908
Male	10 (71.4)	18 (64.3)	
Disease duration (months)	30 (2–72)	24 (2–84)	0.398
Stage			
0	–	7 (25.0)	0.231
1	6 (42.9)	10 (35.7)	
2	4 (28.6)	6 (21.4)	
3	2 (14.3)	4 (14.3)	
4	2 (14.3)	1 (3.6)	
DC			
Negative	8 (57.1)	26 (92.9)	0.011*
Positive	4 (28.6)	1 (3.6)	
Weak positive	2 (14.3)	1 (3.6)	
p53			
Negative	3 (50.0)	12 (80.0)	0.401
Positive	3 (50.0)	3 (20.0)	
DEL13q			
Negative	1 (25.0)	4 (40.0)	0.999
Positive	3 (75.0)	6 (60.0)	
Haemoglobin (g/dL)	12 ± 2.2	12.7 ± 2.2	0.327
WBCs (×10^3^/μL)	29.6 (5.8–128.7)	25.3 (6.4–131)	0.539
Neutrophils (×10^3^/μL)	5.6 (0.7–8)	5.5 (0.6–11.5)	0.831
Lymphocytes (× 10^3^/μL)	24.3 (5–123)	17.5 (5.2–116.5)	0.496
Platelets (×10^3^/μL)	200 (81–463)	200 (24–350)	0.800

Numerical variables are presented as mean ± standard deviation or median (min-max) according to normality distribution. Categorical variables are presented as number (%)

* *P* < 0.05 indicates statistical significance

Abbreviations: *CLL* Chronic lymphocytic leukaemia, *WBC* White blood cell

## Discussion

Previous studies have shown that ELA possesses anti-apoptototic activity [15, 19]. Although the role of ELA in cancer has been investigated in a limited number of studies [20, 21], several studies have shown that apelin, which is the other endogenous ligand of APRLN, is overexpressed in many tumour tissues and cell lines, and the apelin/APLNR system plays a role in the regulation of cancer cell growth and migration [22–24].

In the present study, ELA levels were significantly higher in patients with CLL than in control group patients. This finding supports the anti-apoptotic effects of ELA and the apelinergic system reported in the literature.

Seo et al. showed that DNA damage-induced hnRNP L upregulates *p53* expression [25].

Li et al. showed that ELA downregulates the interaction between hnRNPL and *p53* [26], resulting in an anti-apoptotic effect. Additionally, Ganguly et al. reported increased ELA gene expression levels in glioblastoma cells and that an association exists between upregulated expression of ELA and poor prognosis [21]. Yi et al. reported increased ELA expression levels in ovarian cancer cells. Disruption of ELA expression in these cell lines suppressed cell growth, cell migration and cell cycle progression. They showed that ELA exerted this effect independently of APLNR, affecting cell growth and cell cycle progression in a *p53*-dependent manner. Loss of ELA in cells expressing high levels of *p53* caused a decrease in cell number due to cell death, and this resulted from p53-induced cell apoptosis [20]. Mouse double minute 2 (MDM2) is a critical negative regulator of tumour suppressor p53 and plays a key role in controlling its transcriptional activity, protein stability and nuclear localisation. MDM2 expression is upregulated in many cancers, resulting in a loss of p53-dependent activities, such as apoptosis and cell cycle arrest [27]. The PI3K/Akt signalling pathway has been shown to play a critical role in the tumourigenesis of haematopoietic cells. Activation of the PI3K/Akt pathway occurs even in the early stages of tumour development, and it correlates with poor prognosis and therapeutic resistance in various human cancers [15, 28]. ELA activates the PI3K/AKT/mTORC1 signal to promote the progression of hESC cell cycle and protein translation and blocks stress-induced apoptosis. These pathways are the main signals reported to be correlated with apoptosis. MDM2 also inhibits p53 through this pathway. It has been suggested that the apelinergic system may inhibit apoptosis through these common pathways (7–11, 28).

hnRNPC is a negative regulator of p53. A previous study showed that the 1–41 p53 region, which is the region where p53 binds to Mdm2, also interacts with hnRNPC. These results show that hnRNPC may be synergistic with Mdm2 in regulating p53 stability. Doxorubicin competes with p53 for binding to the RNA recognition motif of hnRNPC, thereby enhancing p53 stability and triggering p53-dependent apoptosis [29]. ELA, which has been shown to possess anti-apoptotic activity, has been shown to interact with the CXCR4a signalling pathway, one of the chemokines [15, 19]. Chemokines are produced by cancer-associated fibroblasts, a component of stromal cells, and affect metastatic potential and site-specific spread of cancer cells. The stromal cell-derived factor-1 (SDF-1/CXCL12) belongs to the family of CXC chemokines. The effects of CXCL12 in many cancer types, including its role in promoting local invasion and distant metastasis from lung cancer metastasis, have been described [30–32]. Wang et al. showed that CXCL12 blocks apoptosis in human adenocarcinoma cell line via CXCR4. They observed that the expression levels of Bcl-2 and bcl-xl in the adenocarcinoma cell line increased with CXCL12 therapy and decreased with CXCR4 antagonist and JAK2 inhibitor therapy [33]. In summary, ELA and the apelinergic system have been shown to inhibit apoptosis in several steps (via bcl-2, bcl-xl, mdm2, hnRPLN, p53, and PI3K/Akt/mTORC1). Based on these results, it can be suggested that ELA and the apelinergic system play a central role in the pathogenesis of CLL.

In the present study, we showed that serum ELA levels were significantly high in patients with CLL. This finding indicates that ELA contributes to the development of CLL, which is consistent with the findings of other studies in the literature.

Venetoclax is a bcl-2 inhibitor and idasanutlin is a MDM2 inhibitor, and both are indicated for use in CLL. Venetoclax + idasanutlin have been suggested to be an effective treatment for relapsed/refractory acute myeloid leukaemia (AML) [34]. However, inhibition of ELA or the apelinergic system will exert the effect of both venetoclax and idasanutlin. In other words, inhibition of the apelinergic system alone can provide a treatment as effective as venetoclax and idasanutlin or even a combination of the two. Yi et al. showed that human ELA can downregulate p53 protein levels and activity in cancer cells instead of working as a p53 activator. Although ovarian cancer cells are typically normal type p53, no studies have assessed whether there is a correlation between p53 mutation status and ELA expression levels in ovarian cancer [20].

Because the number of patients with *TP53* mutation was insufficient in the present study, we could not perform a statistically significant evaluation. However, future studies evaluating a sufficient number of patients will be valuable for CLL, in which *TP53* mutations occur frequent. The results of our study provide evidence that ELA and the apelinergic system can be valuable in targeted therapy and may also be useful in predicting patient prognosis, response to treatment and follow-up. More comprehensive studies are needed to address these issues.

## Conclusions

This study highlights the effects of ELA on CLL and emphasizes that ELA targeting may be a potential therapeutic option for treating CLL.

## Data Availability

The datasets generated for this study are available from the corresponding author on reasonable request. The authors declare that all other data supporting the findings of this study are available within the article and its Supplementary Information Files. Additional files.

## Abbreviations

CI: Confidence intervals
CLL: Chronic lymphocytic leukemia
ELISA: Enzyme-linked immunosorbent assay
Hb: Hemoglobin
OR: Odds ratio
PLT: Platelet
WBC: White blood cell count
